# Neonatal pneumothorax from the perspective of a pediatric surgeon: classification and management protocol: a preliminary algorithm

**DOI:** 10.3906/sag-2010-286

**Published:** 2021-06-28

**Authors:** Mirzaman HUSEYNOV, Ali Ekber HAKALMAZ

**Affiliations:** 1 Department of Pediatric Surgery, Private Safa Hospital, İstanbul Turkey; 2 Department of Pediatric Surgery, Private Avicenna Hospital, İstanbul Turkey; 3 Department of Pediatric Surgery, Gaziosmanpaşa Training and Research Hospital, İstanbul Turkey

**Keywords:** Newborn, pneumothorax, thoracostomy, classification, management

## Abstract

**Background/aim:**

Current neonatal pneumothorax classifications based on air volume escaping in pleural space have no contribution on the treatment. Therefore, our aim was to classify neonatal pneumothorax to guide treatment management based on our experiences.

**Material and methods:**

The records of all neonates admitted to our clinics from March 2017 to August 2020 were reviewed. The patients with pneumothorax were identified through the neonatology department patient database search. The study only included the patients with symptomatic pneumothorax and these patients were evaluated into 3 groups based on the changes in peripheral oxygen saturation (SpO2) and clinical features immediately after the tube thoracostomy (TT) procedure. Accordingly, neonatal pneumothorax was divided into 3 types: patients with SpO2 increasing immediately after TT were included in type I, patients whose SpO2 did not change after TT were included in type II, and patients with SpO2 decreasing immediately after TT were included in type III pneumothorax.

**Results:**

A total of 82 patients were included in the study. Sixty-one percent of these patients had type I, 24% had type II, and 15% had type III pneumothorax. None of the neonates died in type I and II pneumothoraces while 9 of 12 neonates (75%) died within the neonatal period in type III pneumothorax. Although we applied treatments such as high-frequency oscillatory ventilation, selective intubation, continuous negative aspiration, and surgical treatment to our patients that were lost due to type III pneumothorax, we were not successful. We successfully managed our surviving type III pneumothorax patients with a simple pressure cycle ventilator, using a combination of high rates, modest peak airway pressures [18 to 22 cm H2O and no positive end-expiratory pressure (PEEP)], and an autologous blood patch.

**Conclusion:**

Classification of pneumothoraces into different types significantly contributes to patient treatment planning through a predetermined strategy, not through trial-and-error. High frequency and zero PEEP ventilation can provide significant improvement in risky cases.

## 1. Introduction

As the most common air leakage type, pneumothorax is defined as air accumulation within the pleural space [1]. Pneumothorax develops in up to 1.5%–7% of neonates [2]. The incidence of pneumothorax increases dramatically in the presence of a lung disease, especially in need of mechanical ventilation. Bilateral pneumothoraces constitute fifteen to twenty percent of all pneumothoraces [3]. Two-thirds of unilateral pneumothoraces affect the right side. Affected neonates may be asymptomatic or have various degrees of respiratory distress. Diagnosis is suspected clinically because of respiratory distress and increased oxygen requirement and confirmed by X-ray.

There are a few classifications for neonatal pneumothorax in the literature, all of which are prognosis-oriented and are based on air volume escaping into the pleural space [3,4]. To the best of our knowledge, there is no clinical classification or algorithm that can guide the management of neonatal pneumothorax cases currently. Treatment protocols are well defined based on pneumothorax volume in adult series [5]. However, adult protocols are difficult to adapt to newborns, and their applicability is controversial. Therefore, it is essential to establish a classification for newborns to guide clinicians. This study aims to classify neonatal pneumothoraces to provide appropriate management approaches.

## 2. Material and methods

### 2.1. Study population and inclusion/exclusion criteria

This is a retrospective descriptive study. After obtaining permission from the local authority (no.: 2020–125), the records of all neonates admitted to our clinics from March 2017 to August 2020 were reviewed. The patients with pneumothorax were identified through neonatology department patient database search. The hospital charts and chest X-rays were obtained. All investigations were reviewed by a radiologist and a pediatric surgeon. Only patients with symptomatic pneumothorax were included in the study while newborns with major cardiac anomalies, renal failure, and other known causes of SpO2 decrease (such as congenital diaphragmatic hernia) were not included. All of the patients with pneumothorax were treated by the same neonatologist and pediatric surgeon.

### 2.2. Independent variables and outcomes of interest

Patients were evaluated for baseline characteristics (gestational age, sex, birth weight, and type of delivery), underlying pulmonary conditions [respiratory distress syndrome (RDS), transient tachypnea of the newborn (TTN), and pneumonia], pneumothorax occurrence time, treatment [drainage which consisted of needle aspiration, needle aspiration followed by tube thoracostomy (TT) or TT alone, double/triple TT, continuous aspiration with negative pressure, surgical intervention, and high-frequency oscillatory ventilation (HFOV), autologous blood patch (ABP)], thoracostomy tube removal time, and outcomes (survived or died).

### 2.3. Pneumothorax management protocol of our unit

The indication for drainage of pneumothorax in our unit is determined by the neonatologist. The pediatric surgeon meets the patient for the first time during chest drain insertion. Regardless of patient-specific differences, draining was the general pneumothorax management protocol of our unit in the presence of respiratory distress, low SpO2, and/or hemodynamic instability. In the presence of a nonsevere respiratory distress or a slight SpO2 decrease and the lack of a severe hemodynamic instability, needle aspiration was attempted first and if air kept on accumulating, TT was performed. All procedures were performed following the standard operating procedure guidelines for chest drain insertion [6]. In accordance with the neonatal intensive unit routine procedure, parents were informed about the chest tube insertion as soon as possible. However, written informed consent was not routinely sought due to the usual emergency of this procedure.

### 2.4. Definitions and settings

Neonatal pneumothorax was defined as pneumothorax verified by chest radiography up to 28 days of life. Low SpO2 was defined according to the ‘’Clinical recommendations for SpO2 targeting in various neonatal conditions’’ [7]. Although the initial mechanical ventilation settings in intubated patients vary according to the patient characteristics, they were generally made according to the ‘’Clinical recommendations for SpO2 targeting in various neonatal conditions’’ [7]. The success of the TT procedure was defined as the complete radiological resolution of the pneumothorax after drainage.

The patients were divided into 3 groups according to the change in SpO2 (increased, decreased, and unchanged) and clinical features immediately after the TT procedure. Accordingly, neonatal pneumothorax was divided into three types: patients with SpO2 increasing immediately after TT were included in type I, patients whose SpO2 did not change after TT were included in type II, and patients with SpO2 decreasing immediately after TT were included in type III pneumothorax.

## 3. Statistical analysis

Statistical analyses were performed using the SPSS software v. 22.0 (SPSS Inc., Chicago, IL, USA). Normal distribution was assessed through the Kolmogorov–Smirnov test. The homogeneity of variance was determined by the Levene test. Parametric variables were analyzed using the one-way ANOVA test and nonparametric variables with the Kruskal–Wallis test. To compare qualitative variables between groups, the chi-square test with Fisher exact test correction was used. The level of statistical significance was set at p < 0.05.

## 4. Results

During the study period, 3600 neonates were admitted to the NICU. Neonatal pneumothorax was diagnosed in 82 patients. The incidence of pneumothorax among hospitalized neonates was 2.3%.

Clinical and demographic data for neonates with pneumothorax are shown in Table. Males outnumbered females by a ratio of 2.6:1. Pneumothorax was on the right in 42 patients, on the left in 20, and bilateral in 20 patients. The mean gestational age (GA) ± SD was (31 + 2) ± (5 + 2) weeks, and the mean birth weight ± SD was 1804.4 ± 982.6 g. Sixty-two percent of the neonates were delivered by cesarean section. All patients had an underlying pulmonary disease, predominantly RDS (60%) and TTN (26%).

**Table:** Demographic characteristics of patients, clinical variables, and their statistical significance.

	Total	Type I	Type II	Type III	p
SexMale	62	38	17	7	
Female	20	12	3	5	
GAMean (range - /- )	31 + 2(21 + 5/39 + 2)	32 + 6(25 + 3/39 + 2)	32+6(26 + 3/37 + 4)	26 + 3(21 + 5/34)	0.000
Birth weight Mean (range)LBWVLBWELBW	1804.40(330–3610)33912	2473.5(685–3610)2231	2061.6(970–2835)1042	895(330–2280)129	0.0000.040.1890.000
Diagnosis TTN	21	18	3	0	
RDS (surfactant)	49	24 (23)	14 (13)	11 (11)	0.024
CP	12	8	3	1	
Type of deliveryNSD	21	14	3	4	
CS	61	36	17	8	
LateralityRight	42	28	8	6	
Left	20	8	9	3	0.041
Bilateral	20	14	3	3	
POT (days)Mean (range)	3.4(0–27)	1.14(0–4)	3.6(3–12)	6.3(0–27)	
LST (days)Mean (range)	7.2(2–20)	6.9(2–16)	12(7–18)	15.6(12–20)	0.040
EXYes	9	0	0	9	
No	73	50	20	3	

GA, gestational age; LBW, low birth weight; VLBW, very low birth weight; ELBW, extremely low birth weight; TTN, transient tachypnea of the newborn; RDS, respiratory distress syndrome; NSD, normal spontaneous vaginal delivery; CS, cesarean section; POT, pneumothorax occurrence time; LST, length of stay of the chest tube.

The median age at diagnosis of pneumothorax was 3.4 days (range: 0–27 days). Needle aspiration followed by chest drain placement was carried out in 5 neonates, while 77 patients only had a chest drain. The mean length of stay of the chest tube was 7.2 days (range: 2–20 days). Nine neonates died within the neonatal period, corresponding to a mortality rate of 11%.

Pneumothorax was divided into 3 types according to the change in SPO2 and the clinical features immediately after TT:

### 4.1. Type I pneumothorax (61%) (Figure 1)

**Figure 1:**
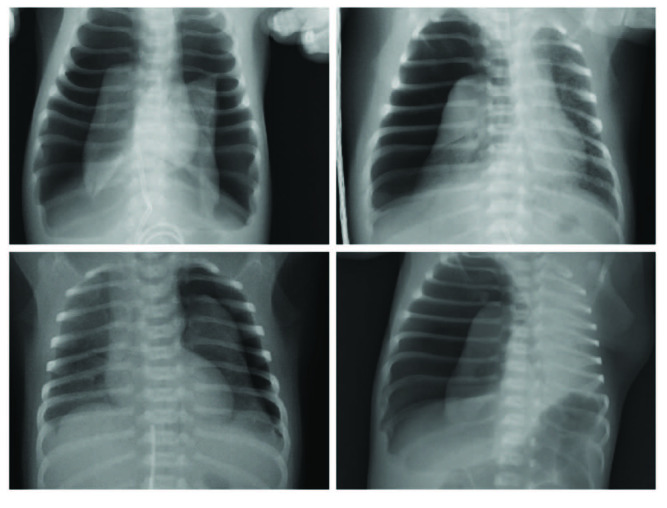
Radiographs of patients with type I pneumothorax.

Patients with SpO2 increasing immediately after TT were included in this group (n = 50). Clinical and demographic data of these patients are shown in Table. Of affected neonates, 32 (64%) were preterm. All patients had an underlying pulmonary disease, predominantly RDS (48%) and TTN (36%). 

Five of these patients were taken to continuous aspiration with negative pressures of 5–10 cm H2O since SpO2 did not return to normal levels despite the increase in SpO2. In these patients, pneumothorax was observed to continue on chest radiographs, despite the decrease. Persistent pneumothorax developed in 3 of the patients (6%) in whom autologous blood patch was successfully applied. None of the neonates in this group died.

### 4.2. Type II pneumothorax (24%) (Figure 2)

**Figure 2:**
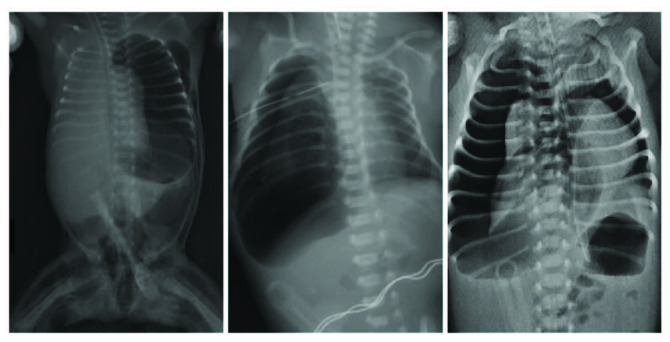
Radiographs of patients with type II pneumothorax.

**Figure 3:**
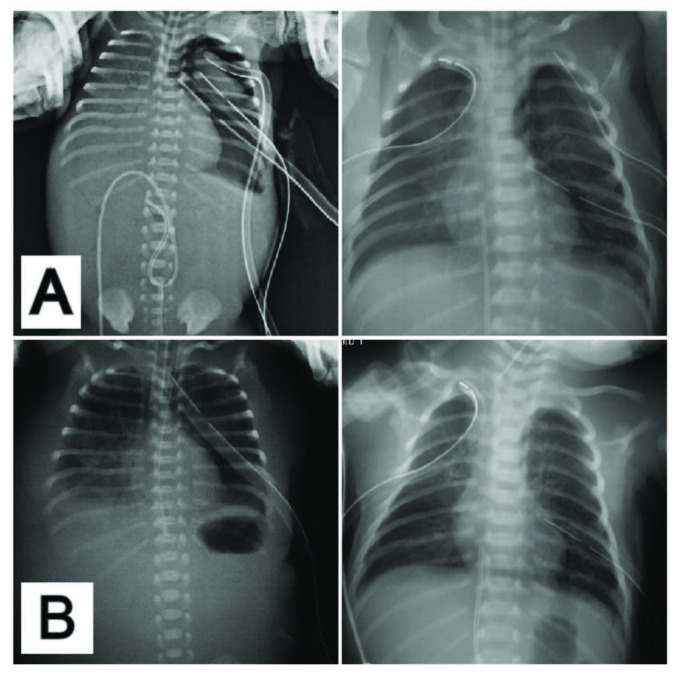
A) Patients with double and triple chest drain, B) same patients after continuous negative aspiration.

Patients whose SpO2 did not change after TT were included in this group (n = 20). Since the saturation did not change and the pneumothorax persisted in the radiographs, all patients were taken to continuous aspiration with negative pressures of 5–10 cm H2O. A second thoracic drain, which was previously inserted by a neonatologist in 4 patients, was removed and negative aspiration was applied to these patients also (Figures 3A and 3B). Saturations returned to normal in all patients after negative aspiration. However, persistent pneumothorax developed in 3 (15%) patients in whom autologous blood patch was successfully applied.

Clinical and demographic data of these patients are shown in Table. Of affected neonates, 16 (83%) were preterm. All patients had an underlying pulmonary disease, predominantly RDS (70%) and TTN (15%). None of the neonates died in this type of pneumothorax.

No statistically significant difference was found in sex, gestational age, underlying pulmonary disease, surfactant therapy use and delivery type between type I and type II pneumothoraces. Left-sided pneumothorax was statistically more common in type II patients (p = 0.010). No statistically significant difference was found between patients with type I and type II pneumothoraces in terms of the development of persistent pneumothorax (p = 0.735). 

### 4.3. Type III pneumothorax (15%) (Figure 4)

**Figure 4:**
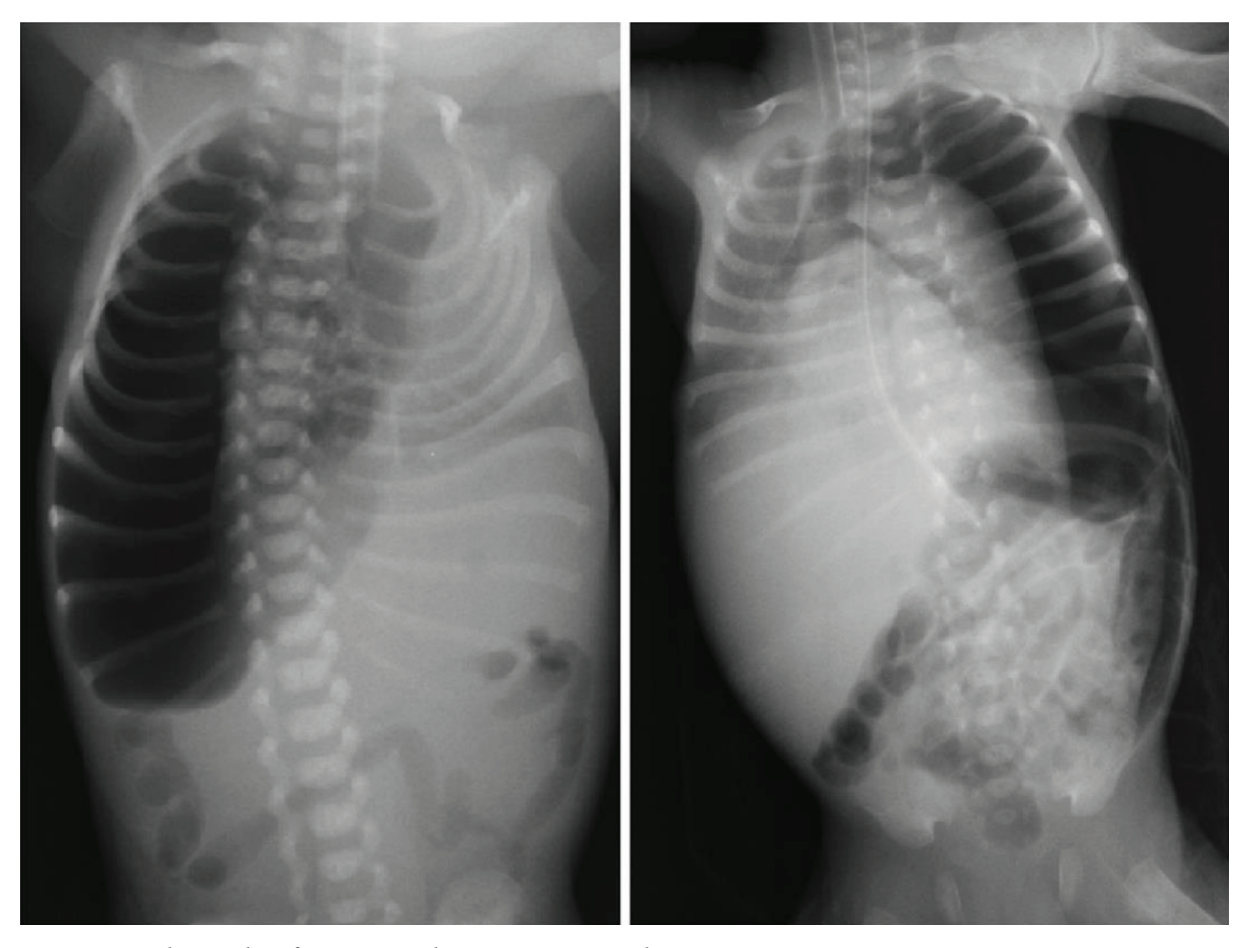
Radiographs of patients with type III pneumothorax.

Patients with SpO2 decreasing immediately after TT were included in this group (n = 12). Immediately after chest drain insertion, patients’ saturation decreases even more with a very rapid decompensation (death occurred within 3 h after TT). Clinical and demographic data of these patients are shown in Table. All affected neonates were preterm. GA was the main feature of these patients with birth weights lower than the previous groups (p = 0.000). All patients had RDS except for one. RDS was significantly higher in patients with type III pneumothorax patients compared to other groups (p = 0.024). Nine of the patients died in this type of pneumothorax (75%). 

In this group, to the patients that were lost, HFOV and continuous negative aspiration (to all of them), trying to selectively intubate the opposite main bronchus (in 4), drain clamping (in 1), emergency operation (in 2), Fogarty catheter application (in 2) were applied. In the surviving type III pneumothorax patients, simple pressure cycle ventilator, using a combination of high rates, modest peak airway pressures [18 to 22 cm H2O and no positive end-expiratory pressure (PEEP)], and an autologous blood patch treatment was applied following the unsuccessful application of HFOV and continuous negative aspiration. 

There was no statistically significant difference between the groups in terms of the number of very low birth weight patients. The number of patients with extremely low birth weight was statistically higher in type III pneumothorax group.

## 5. Discussion

Pneumothorax is a common problem in neonates taking respiratory support [2]. To the best of our knowledge, no standard classification or treatment management algorithm is yet available for neonatal pneumothoraces. Our study is the first and only study in the literature classifying neonatal pneumothorax according to clinical findings and SpO2 course and also providing treatment suggestions in line with this classification. In addition, it may interest clinicians as it offers a new perspective on unresolved situations in persistent air leakage with poor prognosis. The need for approaches with a high success rate is obvious in these cases [8,9].

Pneumothorax classifications in the literature were generally inspired by adult pneumothorax cases and were based on radiologically detectable pneumothorax volume [10,11]. Özer et al. claimed in their study that this calculation method could be applied to neonates [4]. They found that the calculation of pneumothorax size revealed statistical significance as a prognostic factor in the neonatal period, the pneumothorax size was significantly larger in nonsurvivors and appeared as an independent prognostic factor (>20%). In conclusion, they argued that identifying the risk groups by calculation of pneumothorax size can be a practical and reliable method. However, in our series, the number of patients who have a similar pneumothorax size but give different responses to the treatment was not very low. Figures 1, 2, and 4 provide examples of some of these. Additionally, many variables such as parenchymal disease or diagnosis time may cause a clinically significant change in pneumothorax volume. The number of cases with such variables is higher in practice. Moreover, the treatment suggestions based on these classifications do not cover posttube thoracostomy period. Consequently, the available classifications have a quite limited applicability. We think that the approaches for airway injuries should be applied for persistent pneumothorax.

**Figure 5:**
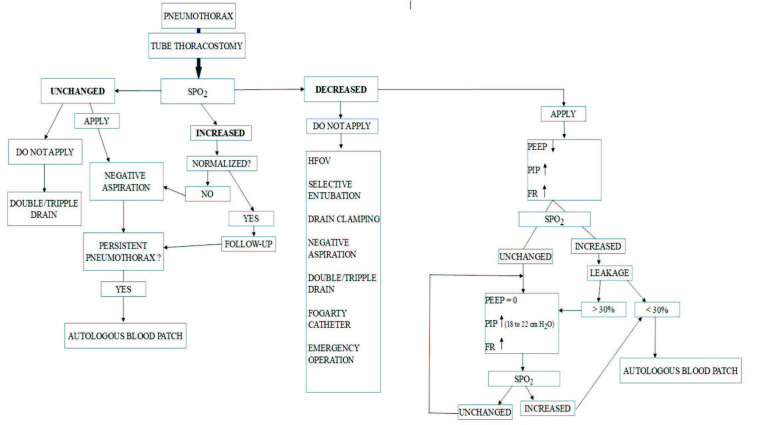
Proposed preliminary algorithm for the management of neonatal pneumothoraces. (HFOV, high-frequency oscillatory ventilation; PEEP, positive end-expiratory pressure; PIP, peak inspiratory pressure; FR, frequency).

Supportive treatments may be successful in pneumothoraces lacking hemodynamic and respiratory instability, including symptomatic ones [12,13]. Needle aspiration or TT alone is adequate in many cases requiring intervention [14]. An additional intervention was not required in 60% of the pneumothorax cases irresponsive to needle aspirations and requiring TT in our series. On the other hand, queries are still present for the management of 40% of the cases not regressing and even worsening despite TT. The algorithm we suggested may help clinicians in the management of neonatal pneumothoraces covering such difficult cases and may inspire new studies (Figure 5).

Air leakage continuing after TT is an important problem which increases hospitalization, mechanic ventilation, morbidity, and mortality [9,12,15]. In patients without worsening of oxygenation findings after TT (type I and type II), negative aspiration should be the next step for ongoing air leak [16]. We do not recommend the insertion of a second or third chest drain in these patients since our observations suggested that continuous negative aspiration with appropriate pressure was more effective than additional tube thoracostomies (Figure 3). Moreover, the application of continuous negative aspiration instead of the second or third chest drain prevents cosmetic problems and possible complications related to the application. Air leakage recovered through negative aspiration in 19 out of these 25 patients in our series (76%). However, persistent pneumothorax developed in 6 of the patients. ABP, povidone-iodine, and fibrin glue application were suggested for these patients in the literature [17–19]. Complete recovery was provided through ABP technique without any complications in our cases.

In practice, the biggest problem is experienced with the patients we classified as type III pneumothorax. No recommended treatment options are available for such patients in the literature. In such patients, a clinician may sometimes lack adequate time to recognize and treat the airway injury. Operations including bronchoscopy, selective intubation, and surgical intervention can only be applied under sustainable respiratory conditions [20]. Time-gaining, fast, and effective steps are required to provide these conditions. However, quite limited actions can be done in centers lacking extracorporeal membrane oxygenation (ECMO) as a means to provide cardiorespiratory stabilization in these cases, which also have parenchymal disorders [21].

Selective intubation or occlusion constitutes methods with previously reported positive results in persistent air leakage cases [22,23]. However, selective bronchial occlusion or intubation is a technically difficult approach and causes a significant waste of time. Unfortunately, all the patients whom we tried to intubate or occlude the left lung with the Fogarty catheter were lost. Since patient saturation decreases even more, the patients decompensate very rapidly and need urgent interventions after chest drain insertion; moreover, we do not recommend selective intubation or occlusion.

As in some persistent pneumothorax cases, type III pneumothorax may be caused by a defect in the parenchyma or tracheobronchial tree that is too large to heal spontaneously. Therefore, at first glance, surgical closure of this defect may seem like a reasonable approach to the surgeon. Although surgery is a recommended method, especially in persistent pneumothorax cases [23], we think that it should not be preferred in type III patients. This is due to the fact that the operation preparation, unfortunately, causes a severe time loss in these patients competing with time. In our series, two of our patients underwent emergency surgery but died before opening the thorax as a result of rapid decompensation.

HFOV has a controversial place in neonatal pneumothorax cases. Some authors reported better results for HFOV in pneumothorax development and persistent air leakage compared to conventional mechanical ventilation (CMV) [24–27]. However, important studies and metaanalyses showed CMV to be as efficient as HFOV in neonatal air leakages and HFOV was even related to persistent air leakages [13,28]. HFOV was applied to all type III pneumothorax patients who died in our series, and the settings were kept at the recommended level in the literature [29]. However, this application did not reduce leakage, cause an increase in saturation, or prevent the progress of decompensation in any of the patients. We think that HFOV should be applied for hemodynamically stable patients and not be used in type III pneumothorax cases.

High frequency in conventional mechanical ventilation was shown to be safer in terms of pneumothorax formation [30]. However, no clearly suggested ventilation strategy is yet available in high-risked cases especially with massive air leakage in pneumothoraces. We successfully managed our surviving type III pneumothorax patients with a simple pressure cycle ventilator, using a combination of high rates, modest peak airway pressures (18 to 22 cm H2O, and no PEEP), and ABP. Considering that type III pneumothorax is caused by a large parenchymal or tracheobronchial defect and that the main purpose of ventilation is to keep functional respiratory capacity higher, we can conclude that the ventilation strategies of the patients should be similar to those of tracheobronchial injuries and congenital diaphragmatic hernias. The key points in CMV in tracheobronchial injuries and congenital diaphragmatic hernias are low pressure, higher frequency, low tidal volumes, and avoidance of PEEP [31–33]. These approaches aim to reduce barotrauma and keep functional respiratory capacity higher. Consequently, in our surviving patients, we used a combination of high rates and modest peak airway pressures (18 to 22 cm H2O, and no PEEP). The lowest frequency was used to keep SpO2 above 85%. This strategy provided more effective ventilation compared to other interventions and HFOV.

In surviving type III pneumothorax patients, we applied the ABP technique after the leakage in the ventilator decreased to less than 30% (pleurodesis attempts were unsuccessful in case of a leakage over 30%). Autologous blood patch pleurodesis was shown to be a simple, successful, and safe procedure in the adult population. This technique has recently been used safely in the pediatric population [23,34]. In our opinion, this procedure is safe and effective when performed with the correct technique and can also be used safely in newborns. We use this technique safely in our newborn patients and we have not encountered any complications so far [17].

The retrospective nature and insufficient number of patients are the main limitations of the study.

In conclusion, classification of pneumothoraces into different types and determination of treatment management according to this classification, as we recommend, make a great contribution to the planning of the treatment of patients through a predetermined strategy, not by trial and error. In our opinion, this will avoid unnecessary applications in patients who are hemodynamically unstable and decompensate rapidly. High frequency and zero PEEP ventilation can provide significant improvement in risky cases.

## Funding

No financial or nonfinancial benefits have been received or will be received from any party related directly or indirectly to the subject of this article.
